# Genetic testing for breast cancer risk, from *BRCA1/2* to a seven gene panel: an ethical analysis

**DOI:** 10.1186/s12910-020-00545-8

**Published:** 2020-10-21

**Authors:** Erik Gustavsson, Giovanni Galvis, Niklas Juth

**Affiliations:** 1 Division of Society and Health, Department of Health, Medicine and Caring Sciences, Linköping University, 581 83 Linköping, Sweden; 2 Centre for Applied Ethics, Department of Culture and Society, Linköping University, Linköping, Sweden; 3 LIME, Stockholm Centre for Healthcare Ethics, Karolinska Institutet, Stockholm, Sweden

**Keywords:** Genetic testing, Breast cancer, Ethics, Secondary findings, Variants of uncertain significance

## Abstract

**Background:**

Genetic testing is moving from targeted investigations of monogenetic diseases to broader testing that may provide more information. For example, recent health economic studies of genetic testing for an increased risk of breast cancer suggest that it is associated with higher cost-effectiveness to screen for pathogenic variants in a seven gene panel rather than the usual two gene test for variants in *BRCA1* and *BRCA2*. However, irrespective of the extent to which the screening of the panel is cost-effective, there may be ethical reasons to not screen for pathogenic variants in a panel, or to revise the way in which testing and disclosing of results are carried out.

**Main text:**

In this paper we discuss the ethical aspects of genetic testing for an increased risk of breast cancer with a special focus on the ethical differences between screening for pathogenic variants in *BRCA1/2* and a seven gene panel. The paper identifies that the panel increases the number of secondary findings as well as the number of variants of uncertain significance as two specific issues that call for ethical reflection.

**Conclusions:**

We conclude that while the problem of handling secondary findings should not be overstated with regard to the panel, the fact that the panel also generate more variants of uncertain significance, give rise to a more complex set of problems that relate to the value of health as well as the value of autonomy. Therefore, it is insufficient to claim that the seven gene panel is preferable by only referring to the higher cost effectiveness of the panel.

## Background

### Introduction

Traditionally genetic testing to estimate increased lifetime risk of breast cancer (BC) has focused on pathogenic (as opposed to benign) variants in the genes *BRCA1* and *BRCA2*. The development of new genetic technologies allows the identification of several breast cancer predisposition genes (gene-panels). These multiple gene panels include the analysis in known genes such as *BRCA1/2* as well as other genes that are associated with an increased risk for BC. Recent health economic studies suggest an association with higher cost-effectiveness (primarily in terms of extended life expectancy) to screen for pathogenic variants in a seven gene panel rather than the usual *BRCA1*/*2* test [1, 2]. If screening for pathogenic variants in the seven gene panel is more cost-effective than in *BRCA1/2,* this provides a reason for implementing the former rather than the latter as that would result in more total health for a given resource [3]. However, irrespective of the extent to which screening of the seven gene panel is cost-effective, there may be ethical reasons for not using a multiple gene panel, or to otherwise revise the way in which the testing and disclosing of results are carried out.^1^

In this paper we shall explore the ethical differences between screening for pathogenic variants in a panel of genes as compared to screening for such variants in *BRCA1/2*. Whereas we shall focus on this particular case it is an example of a broader trend in genetic testing, where we increasingly move from very targeted investigations of monogenetic diseases to a broader testing that can give more information but also more information of questionable value, such as secondary findings and/or clinically uncertain information. Therefore, our conclusions may have implications that go beyond only testing for the risk of BC.

We will commence by considering a number of problems arising from *BRCA1/2* testing and from panel testing. Although these problems may arise for both kinds of testing, there may be differences in degree between these testing approaches. Secondly, we investigate the ethical problems specific to cascade screening (as opposed to population screening), which are also common to *BRCA1/2* testing and multiple gene panel testing. Finally, we consider several salient ethical differences between *BRCA1/2* testing and multiple gene panel testing.

### Preliminaries

In order to meaningfully discuss these issues, we first need to ask on what basis (norms and values) such genetic testing can be motivated. Drawing on Juth & Munthe [5], we shall assume that genetic testing can be justified according to two objectives, namely (a) the extent to which such testing contributes to an increased health, quality of life and/or longevity (shortened "health" in the following), and (b) the extent to which such testing can promote an individual's autonomy, i.e. the ability to live in accordance with one’s own preferences, beliefs, plans, etc.[6–8]. These objectives are the ones commonly invoked in the area of genetic screening. Although others are conceivable, such as quality of life in general, they have not been brought up in this discussion. We will therefore leave them aside.

The former motive is, and has since long been, uncontroversial within the ethics of screening [9]. This means that genetic testing may be motivated with reference to individuals’ health if there are measures that can be taken in order to prevent a condition from developing, for instance, risk reduction strategies (surgical, enhanced screening, pharmacological) or lifestyle changes. The latter motive has been more accentuated when testing for disorders for which there are no or few medical advantages with knowing the risk of onset, e.g. pre-symptomatic genetic testing for Huntington’s disease [6]. There are many different conceptions of autonomy [7]. The conception relevant in this context presupposes that information about probable future health status can be conducive to realizing one's preferences and plans, which, in turn, presupposes that leading more autonomous lives by deciding and acting autonomously to a larger extent is valuable for a person. Therefore, genetic testing may be justified in terms of autonomy, even if there are no risk reduction measures to take. The information generated from the test may serve as a basis for decisions according to one’s preferences and life plans. However, when testing for the risk of onset (as in the case of genetic testing for BC) rather than for confirming a diagnosis, considerations of autonomy have been considered important. This is so, since when confirming a diagnosis, the point of departure is that a person has already decided that she wants to find out the explanation of her symptoms. However, when testing for the risk of onset, it cannot be taken for granted that the person taking the test (henceforth “index-person”) wants to find out secondary findings indicating the risk of other potential diseases/conditions, syndromes or findings for which the clinical relevance is uncertain. This consideration of autonomy has a close relation to the right not to know [5, 6] which will be further considered below.

## Main text

### The relevance of how genetic testing is organized

#### Ways in which individuals may take a genetic test

There are at least four ways in which an individual may encounter a genetic test for BC. These encounters may take the form of “screening” or “testing”. Following Juth & Munthe [5] we shall understand the difference between the two by appealing to by whom the test is initiated. There are two forms of screening which are initiated by someone other than the index-person, namely population screening and cascade screening. (a) Population (genetic) screening is a screening program which usually targets people of certain age range within a country’s or region’s population. (b) Cascade screening is a more targeted form of (genetic) screening, where individuals with a recognizably higher genetic risk for a specific disease are offered to take a test, for example, when a test is being offered to other at-risk relatives where a pathogenic variant has been identified in the index-person. Cascade screening is therefore different from population screening but shares the feature that the initiative to testing comes from someone other than the index-person. Accordingly, we shall take a defining characteristic of the notion of screening (population as well as cascade) to be that testing is initiated by the health care sector rather than the individual herself.

Furthermore, *BRCA1/2* testing or multiple gene panel testing is sometimes initiated by the individual (in consultation with health care professionals) for two typical reasons: (c) by an individual who is concerned with the number of cases of cancer in the family (this may aim to assess the risk of healthy family members developing a particular type of cancer), or (d) by an individual with a form of pre-existing cancer in order to provide clinical guidance for this particular case.

Once a pathogenic variant has been detected, however, this generates a risk profile, not only for the index-person, but also for his or her relatives. If, and if so how, this risk profile should be communicated to relatives is a contentious issue [10].^2^ A genetic test initiated by the index-person need not be preceded by worries about his or her family history but may also be motivated by a desire for reassurance that one is not at an increased risk. However, given that relatives are approached, we have turned the issue to one about screening or, more specifically, cascade screening.

The following discussion has relevance for these four kinds of testing. This paper focuses on predictive testing of adults and consequently leaves other types of genetic testing (such as prenatal genetic testing) aside in the following.

### The ethical relevance of the different ways in which people are approached

The distinction between testing and screening has an ethical importance from the point of view of autonomy. In ordinary health care, the individual seeks health care to have a problem remedied. This is at the initiative of the person herself. All follow up testing and treatment are supposed to be decided upon in a dialogue with the patient according to that patient’s needs and desires [7]. In population screening, contrarily, there is a standard testing offered to all within the screening program. Besides the challenge of tailored screening to the individual patient’s needs and desires, this carries with it some pressure to accept the offer to start with: why would testing be offered unless there is a reason to accept it (the approached person is likely to think)?

In cascade screening, the situation is analogous to population screening in the sense that relatives have not themselves approached health care to find out their risk of BC. This is, then, a disadvantage in terms of autonomy compared to the ordinary situation where the individual takes the initiative to seek out health care. Moreover, there are some downsides with cascade screening from the point of view of autonomy as such screening differs from population screening as the risk, quite naturally, is higher. This is the defining characteristic of the targeted population, because of their increased risk based upon the fact that one of their relatives had tested positive for a pathogenic variant.

### *BRCA1/2* testing versus panel testing: common problems

Any screening, whether it is genetic or not, uses some method of risk stratification in order to detect suitable candidates for further testing and/or intervention. Partly because no such method is perfect, test results do not come out with 100% accuracy.

This is also true for genetic screening for pathogenic variants in *BRCA1/2* and panel testing. In a sense, both these tests are very reliable in detecting the mutations targeted (almost 100%). However, since both these tests target pathogenic variants with less than 100% penetrance, some people identified as being high risk-individuals, will never develop BC. This means that although there is an effect in terms of increased life expectancy on a group-level resulting from screening, there is also some degree of overtreatment, i.e. treatment of individuals who would not have been affected by the disease in question before dying of other causes. Overtreatment is a problem to the extent that individuals are undertaking risk reduction measures, for example mastectomy, for findings that would not have otherwise affected the person’s life if it were not for screening.

Patients with pathogenic variants in high and moderate penetrance genes are informed about the options for risk reduction and/or early detection of BC that implies enhanced surveillance with clinical breast examinations, annual mammography and/or MR of the breast. Bilateral mastectomy gives more than 90% risk reduction of developing BC if a pathogenic variant is detected in *BRCA1/2* and at the present time there is also enough evidence to support further consideration for risk reducing mastectomy when pathogenic variants are detected in *CDH1*, *PALB2*, and *TP53* gene [11].

Different studies have shown a rate of mastectomies, between 11–50% [12–14]. In a cohort study of 282 asymptomatic women who are carriers of pathogenic variants in high and moderate penetrance BC genes, 40% underwent bilateral risk reducing mastectomy within a median of 1 year following genetic testing [11].

Note that the problem of overtreatment may be greater in panel testing than in *BRCA1/2*. For instance, bilateral mastectomy is offered to individuals with pathogenic variants found in *BRCA1* (which is associated with 73% increased risk of developing BC by age 80) as well as to individuals with pathogenic variants found in *PALB2* (which is associated with a 50% increased risk of developing BC by age 80) – the latter is detected by the panel but not by standard *BRCA1/2* testing [15]. Therefore, the likelihood of actually developing BC if a pathogenic variant is detected in a non-*BRCA* panel gene is lower than if such a pathogenic variant is detected in *BRCA1/2* (although there are no studies supporting this yet).

It is important to distinguish between overtreatment and treatment due to overdiagnosis, i.e. being identified as a high risk-individual although one will not be affected by the disease in question before dying of other causes. Of course, overdiagnosis may lead to overtreatment. However, overdiagnosis is problematic from an ethical perspective also because people are worried, besides perhaps treated, unnecessarily. To address this problem, it is of great importance how the index-person is approached and informed more specifically. For example, in Swedish current guidelines risk reducing mastectomy is considered medically indicated for individuals with a pathogenic variants in *BRCA1/2* or *TP53* but not for women carrying a pathogenic variants in *ATM*, *CHEK2* and *NBN* [16, 2, p. 62]. For women carrying a pathogenic variant in one of the latter three genes the recommendation is rather enhanced surveillance, for instance, more frequent screening in the mammography program. If an individual belongs to this group which is subject to more intensive monitoring, it is likely that this contributes to worry and anxiety for the individual [17].

Analogously, testing may lead to false reassurance and undertreatment. Of course, since both the *BRCA1/2* testing and the multiple gene panel are very reliable in picking out the variant, there are virtually no false negative in the strict sense of the word: both tests reliably identify the variant. However, there are persons who will be identified as low risks by the tests, but who eventually will develop BC. In this sense, both tests may lead to false reassurance. Here the panel, as it seems, has an advantage in degree compared to *BRCA1/2* testing: the fact that life expectancy is increased with the panel indicates that it to a larger extent captures people at risk for developing BC. So, as is common in screening, there is a trade-off between over- and undertreatment, as well as with false positives and false reassurance. It is a complex matter how to weigh these benefits and burdens to each other [5].

To sum up: *BRCA1/2* testing and panel testing share the problems of overtreatment, overdiagnosis, and false reassurance and potential undertreatment. However, the two first problems are likely to be more elevated with the panel and the last is likely to be more elevated with the *BRCA1/2* testing.

## *BRCA1/2* testing versus panel testing – secondary findings

As mentioned above, recent health economic studies of genetic testing for BC suggest a higher cost-effectiveness to screen for pathogenic variants in a seven gene panel compared to the usual *BRCA1/2* testing [1, 2]. However, regardless of the extent to which the screening of the multiple gene panel is cost-effective, there may be ethical reasons not to screen with the panel. In the following we shall discuss two such potential reasons: (i) to screen with the multiple gene panel increases the number of secondary findings (SF), and (in Sect. 5) (ii) to screen with the panel increases the number of variants of uncertain significance (VUS).

### Different kinds of findings in genetic testing

As the terminology used for different kinds of findings in genetic testing has been widely discussed [18] we shall, in the following, specify how different terms are used in this paper. Let us begin with SF, secondary findings. We take a finding to qualify as a SF when it goes beyond the initial purpose of the genetic test, in the present case the link between pathogenic variants and an increased risk for BC. However, SF should be distinguished from the notion of pleiotropy which is not a *secondary* finding in the pure sense of the word. To exemplify an instance of pleiotropy, consider a pathogenic variant found in *TP53* which increases the risk of developing BC. However, this type of variant also increases the risk of developing other cancer forms such as ovarian cancer and soft tissue sarcoma, among others [19].

Does the increased risk of developing the other cancer forms qualify as a SF or as an instance of pleiotropy? According to the nomenclature employed in this paper this is determined by how the purpose of the test was described in the first place. Although it may seem strange to describe the increased risk for ovarian cancer indicated by a pathogenic variant in *TP53* as a SF from a clinical perspective (it is well known among clinicians that this risk exists) this is not the case from the index-person’s perspective. Accordingly, an underlying assumption for this discussion is that the crucial ethical questions arise in relation to the index-person.

Instances of pleiotropy as well as SF should be further distinguished from findings for which the clinical importance is unclear, i.e. VUS. The test has identified a variant, but whether this variant is pathogenic or benign is unknown. Hence, while SF are defined in relation to the purpose of a given test a VUS is defined in relation to what is known about this particular variant.

### Focusing secondary findings

So, an ethical issue that becomes particularly relevant in testing for pathogenic variants in a panel of genes is how to handle SF [20]. If handling SF in genetic counselling raises ethical challenges this has relevance for the ethical difference between *BRCA1/2* and the panel test. While there are a number of guidelines in place for how SF should be handled in genetic counselling [21, 22] these guidelines are often in relation to screening of Clinical Exome and Genome Sequencing where the number of SF are more numerous than in the panel. In the following we shall discuss a number of ways in which SF could be handled in genetic counselling. In principle, there are three ways of handling this: (a) to *never* inform the index-person about SF, (b) to *always* inform the index-person about SF, and (c) to *sometimes* inform the index-person about SF, for instance when the index-person has agreed to this prior to testing or when the SF informs about a treatable condition (or some combination thereof). As the discussion unfolds, we will note some ethically relevant differences between these options in relation to *BRCA1/2* testing and panel testing.

Note that it is one thing to give such guidance *before* a test is taken and another thing to provide guidance *after* a test has been taken. It is worth noting that, for example European guidelines developed by EuroGentest prescribe that the index-person should get genetic counselling before and after the test [23]. We will refer to this difference as "pre-test counselling" and "post-test counselling" [24].

To avoid problems with SF, a policy could be adopted saying that the genetic counselor *never* informs the index-person about SF, i.e. only informs her about the variants that may be associated with increased risk for BC. However, this option seems problematic from an ethical perspective for at least two reasons. Firstly, there are obvious health related reasons to inform the index-person about high risks of conditions that could be treated effectively. Secondly, this option ignores the possibility that information about SF may contribute to the index-person’s autonomy. This is so, since the index-person may better plan her life according to her desires and plans based on information regarding SF generated by the test, for instance reproductive decisions.

Another possibility would be to *always* disclose information about SF. Traditionally, it is assumed that all information generated by a genetic test should be disclosed to the index-person within genetic counselling [6, p. 92]. This means that the genetic counsellor gives the index-person all information that the test shows, i.e. including information about SF. However, this is problematic from the point of view of autonomy: the genetic counsellor risks bringing unwanted and unwelcome information to the index-person, since SF are by definition unrelated to the purpose of the testing, namely stratifying the risk for BC. The issue of disclosing SF relates to the “right not to know” which have been particularly discussed in relation to genetic testing [25] and refers to the ethical importance of people having control over the extent to which they receive genetic information. Although the right not to know has sparse, if any, legal support in many countries, it is acknowledged as important from an ethical perspective. In ethical discussions this has been primarily discussed in relation to the risk of future illness and the importance of respecting wishes expressed by individuals who do not want to receive such information. The rationale for the right not to know is primarily related to considerations of autonomy [5, 25]. In general, it has been argued that, if anything, the right not to know is stronger than the right to know, analogously to the position that the right to reject health care is stronger than the right to demand certain health care [7]. If these standard views in medical ethics are accepted, the case for not always disclosing SF is buttressed.

The most autonomy friendly option seems to be to let the index-person decide in advance whether or not they want to be informed about SF or not. However, this option involves some difficulties of its own. In general, it may be difficult to decide whether or not one wants to be informed about SF, since it may be unclear what SF may result from the test in question. If there are a large number of SF with different characteristics, it may be difficult for the index-person to comprehend the information. Even though the process of decision-making may be simplified by allowing the index-person to choose between generic categories, such as “only SF of treatable conditions” there may still be differences in degrees regarding, for example, treatability that makes such decisions difficult to make and difficult to understand. However, this problem should not be overstated in this context. The somewhat limited amount of SF that this particular panel test gives rise to [19], makes the option of sometimes informing a reasonable way to handle the situation.

Note that the option of *sometimes* informing implies a rather ambitious genetic counselling. This is in itself a cost that affects the cost-effectiveness analysis. So, the relevant comparison is not between *BRCA1/2* testing and the multiple gene panel, but between *BRCA1/2* testing and the multiple gene panel organized in an ethically defensible way, e.g. including proper pre-test counselling. This means that the information disclosed to the index-person may be both more complex and more extensive with regard to the panel. Furthermore, it is important to note that more information does not necessarily contribute to a person’s autonomy. For information to contribute to his or her autonomy, the index-person must be able to understand, address and make decisions based on the information she receives within genetic counselling [5, pp. 24, 84].

As complexity increases, more requirements may be placed on genetic counselling. Therefore, although testing for the panel rather than *BRCA1/2* is associated only with a slightly higher cost [26], the need for expanded genetic counselling makes screening for the panel compared to *BRCA1/2* more costly. In other words, if panel testing is to be done in an ethically defensible way (involving pre- and post-test counselling) the cost for expanded genetic counselling may make the panel test slightly less cost-effective as a result.

## BRCA1/2 testing versus multiple gene panel testing: variants of uncertain significance

Furthermore, another complexity regarding the panel (compared to *BRCA1/2*) relates to variants of uncertain significance (VUS), a finding for which the clinical importance is unclear. Genetic testing regularly identifies VUS, even in well characterized genes such as *BRCA1*/2 [27]. However, as the number of genes included in the panel increases, the number of identified VUS increases as well [26, 27]. For example, according to Tung et al., when a panel of 25 genes is performed, about 33,2% of the results reported are VUS [28]. Although the possibility of VUS in screening for variants in *BRCA 1/2* poses ethical issues [29], it becomes more pressing regarding testing for the panel as the possibility of identifying VUS increases [26]. How should information about identified VUS be handled in genetic counselling?

### To never inform about VUS

Bjørn Hofmann discusses this delicate issue by drawing on arguments for and against the aforementioned “right not to know” [29].^3^ On the basis of his discussion of these arguments Hofmann concludes that.

“…both the arguments against and for a right not to know do not apply to the case of IFUS [incidental findings of uncertain significance, i.e. VUS]. As shown, there are many reasons for this, but one common underlying factor is that IFUS does not represent knowledge… In the case of IFUS ignorance is bliss” [30, pp. 5–6].^4^

This conclusion regarding informing index-persons about VUS is too hasty. Hofmann employs a sense of ‘knowledge’ according to which a piece of information has to be “accurate and/or actionable” [29]. However, to be informed *that* one carries a VUS in a specific gene must at least count as propositional knowledge. Therefore, Hofmann’s claim that “there is nothing to know” seems incorrect as there is something to know, namely *that* one carries a VUS in a specific gene that *could* be pathogenic or benign. What we do not know in these cases is whether this specific VUS is in fact pathogenic or benign. However, knowing that it could be pathogenic may provide reason for action.

### To always inform about VUS

To what extent can we sketch an argument that supports informing about VUS? It is important to note that the variants that count as a VUS change over time (and different labs may have different VUS rates depending on the tested population). As research in genetics progresses VUS are reclassified, either to pathogenic or benign variants. In relation to the index-person, reclassification may, for instance, be handled by informing the index-person that she or he carries a VUS and encourage this person to contact health care professionals within a few years to see if that particular variant has been reclassified. Alternatively, a database containing all identified VUS may be developed and the individuals in which VUS are identified may be systematically informed if the VUS is reclassified to a pathogenic variant. That increases the chance of improving health, which is an advantage. However, Vos and colleagues suggest that there are reasons to handle information about VUS carefully as nearly 80% of patients with VUS in *BRCA1/2* believed that this finding was connected with a slightly high risk for cancer [30, 31].

### To sometimes inform about VUS

We have argued above that as screening for the panel increases the risk of SF such screening also increases the need for extended genetic counselling. One may suggest that VUS should be handled in the same way. It seems to make perfect sense to inform the index-person about what a VUS is and then ask the index-person in pre-test counselling whether or not she would like to receive this information in the post-test counselling. One can have reason to seek information, even of an uncertain kind.

However, there is probably a limit to what can be managed within genetic counselling. To be informed that one carries a VUS in a gene in which pathogenic variants may have a strong correlation to several severe cancer forms (such as pathogenic variants in *TP53*) may be difficult to handle irrespective of the quality and extensiveness of genetic counselling.

Accordingly, irrespective of how ambitious pre-test counselling is, it still seems as if VUS are especially problematic. Consequently, we believe that Hofmann’s argument suggests something important, namely that VUS are more problematic from an ethical perspective than SF. However, this is not due to a right not to know but because VUS are so complex from an informational point of view that informing about them seems to undermine rather than promote people's autonomy. Accordingly, even if VUS are handled in the same way as SF (by letting the index-person decide in advance if she or he would like to receive information about VUS) there is an autonomy-based argument against screening for the panel rather than the traditional *BRCA1/2*. As stressed above, for information to promote an individual’s autonomy he or she must understand and be able to make decisions on the basis of the information received.

In summary, while autonomy seems to pull against informing the index-person about VUS, considerations about health may pull in the opposite direction. How strong this pull is, more specifically, is dependent on numerous empirical factors about VUS that are beyond the scope of this paper.

## Conclusions

In this paper we have discussed a number of ethical aspects of genetic testing for BC with a special focus on the ethical differences between screening for variants in *BRCA1*/*2* and a seven gene panel. We have argued that (a) the traditional ethical problems with screening (such as overtreatment) is more problematic with regard to the panel, (b) whereas SF may be problematic to handle in some cases this problem should not be overstated. The multiple gene panel discussed here comes with quite limited number of SF which seems manageable in an autonomy friendly way, and (c) the fact that the multiple gene panel generates more VUS than *BRCA1/2* testing is problematic from the point of view of autonomy. The better cost-effectiveness ratio of multiple gene panel screening versus *BRCA1/2* testing may change if genetic counselling can be designed to counter the problems with panel screening. The latter point can be constructed as an argument against the panel compared to the traditional *BRCA1/2* testing. The extent to which the factors highlighted in this article are actually present requires further exploration. More specifically, the effects on the index-person’s autonomy should be investigated from a health economic perspective. Irrespective of the more specific findings from such studies, the discussion in this paper strongly suggests that health economic analysis cannot reasonably be the only basis upon which decisions about what test to implement are made. Decisions based upon such a ground alone could significantly risk ignoring the relevant ethical differences among the different types of testing.

## Data Availability

Not applicable.

## Abbreviations

BC: Breast cancer
SF: Secondary findings
VUS: Variants of uncertain significance

## Footnotes

1 A panel test may contain different combinations of genes [1, 2, 4]. When we refer to “the panel” in the following we are referring to the seven gene panel employed by Li et al. [1] which consists of *BRCA1*, *BRCA2*, *TP53*, *PTEN*, *CDH1*, *STK11*, and *PALB2*. Whereas some companies (e.g. in the US) offer larger panels with 14 genes (see e.g. https://www.invitae.com/en/physician/tests/01202/) they do not make any claims about its cost effectiveness, neither does the move from 7 to 14 genes included in a panel change the more principled questions that we want to discuss in this paper.

2 These issues are beyond the scope of this paper and we shall therefore not take a stand to the ethical questions that follow from this, but they are plenty. First, there is the question about whether or not relatives should be informed at all. Second, there are questions about if and when the index-person should be allowed to veto approaching relatives. Third, there are practical questions about who should bring the information and how. Whether or not we are dealing with *BRCA1/2* testing or panel testing, these issues need to be tackled. We refer the reader interested in these issues more specifically to a number of other publications [5–7, 10].

3 It should be noted that Hofmann explicitly discusses the relevance of *the right not to know* and whether this principle has implications for whether information about VUS should be disclosed or not. A charitable reading of Hofmann should leave the possibility open that he may accept other reasons for disclosing information about VUS.

4 Note that Hofmann uses a slightly different terminology, whereas we refer to “variants of uncertain significance” Hofmann refers to “incidental findings of uncertain significance”. As far as we can see this difference is merely terminological.
